# Supplementary material for: Serving Two Masters: Effect of Escherichia coli Dual Resistance on Antibiotic Susceptibility
Source: Antibiotics (Basel). 2023 Mar 17;12(3):603. doi: 10.3390/antibiotics12030603 (PMC10044975; doi:10.3390/antibiotics12030603)
Supplement: Supplementary file 1 [file antibiotics-12-00603-s001.zip › antibiotics-2237554-supplementary.pdf]

Supplementary Data

Figure S1. Resistance of populations to bacteriophage *T7*. Representative plates showing plaques of (a) iron (III)/phage resistant (b) Phage resistant (c) Ancestor (d) Control and (e) Iron (III) resistant populations. Significantly higher number of plaques were observed on the ancestral population, followed by the control and iron (III)-resistant populations. Bacteriophage T7 plaques are between 1-2mm in diameter at formation (after 2½ - 3 hours of incubation at 37°C). Plaques can expand to 4-6mm in diameter afterwards. Pictures were taken after 12 hours of incubation.

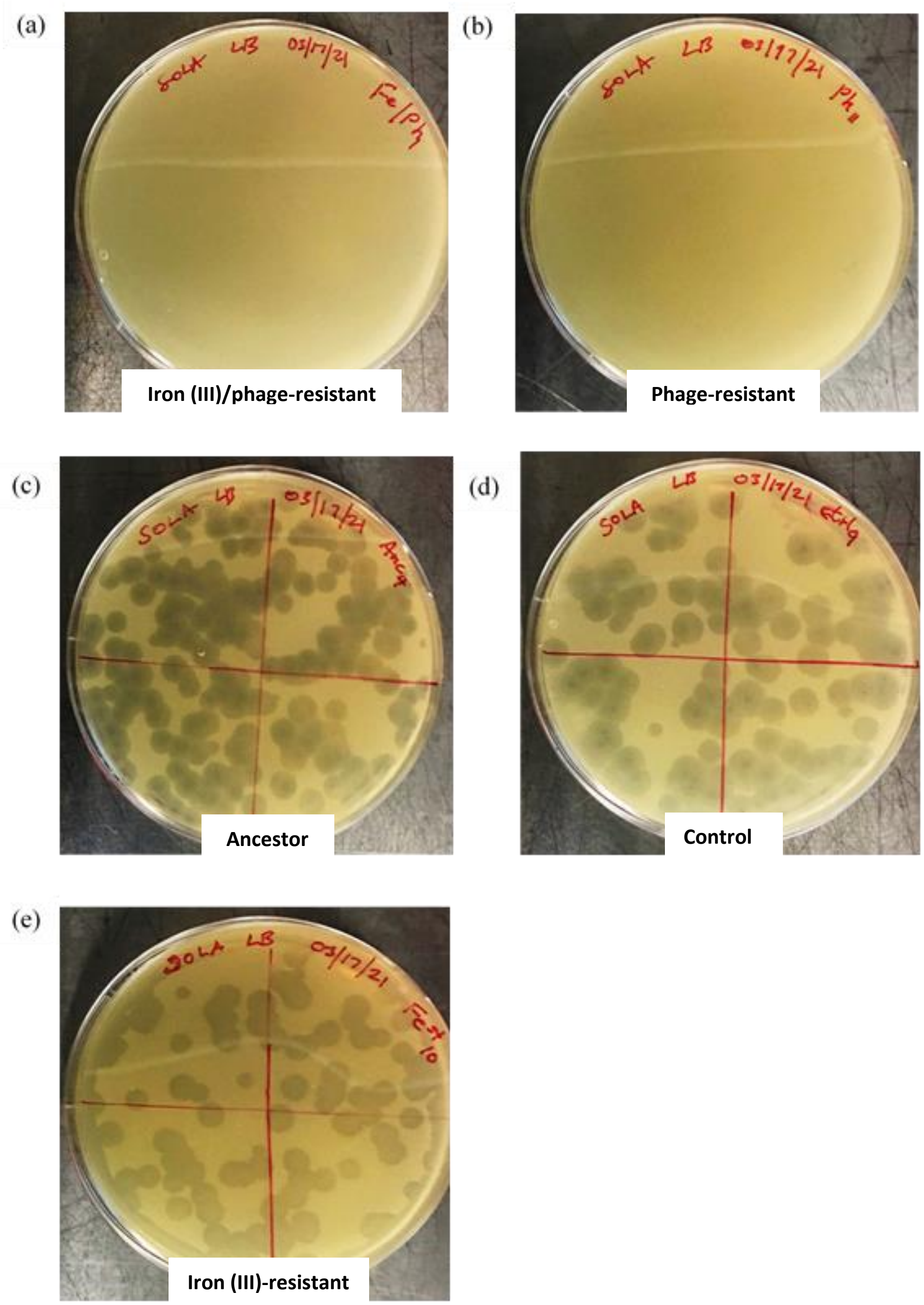

Figure S2. Dual resistance of populations to bacteriophage T7 and iron (III). Representative plates showing bacterial colonies of (a) Iron (III)/phage resistant (b) Control (c) Iron (III) resistant (d) Ancestor and (e) Phage resistant populations. Significantly higher number of resistant bacterial colonies were observed on the ancestral population, followed by the control and iron (III)-resistant populations.

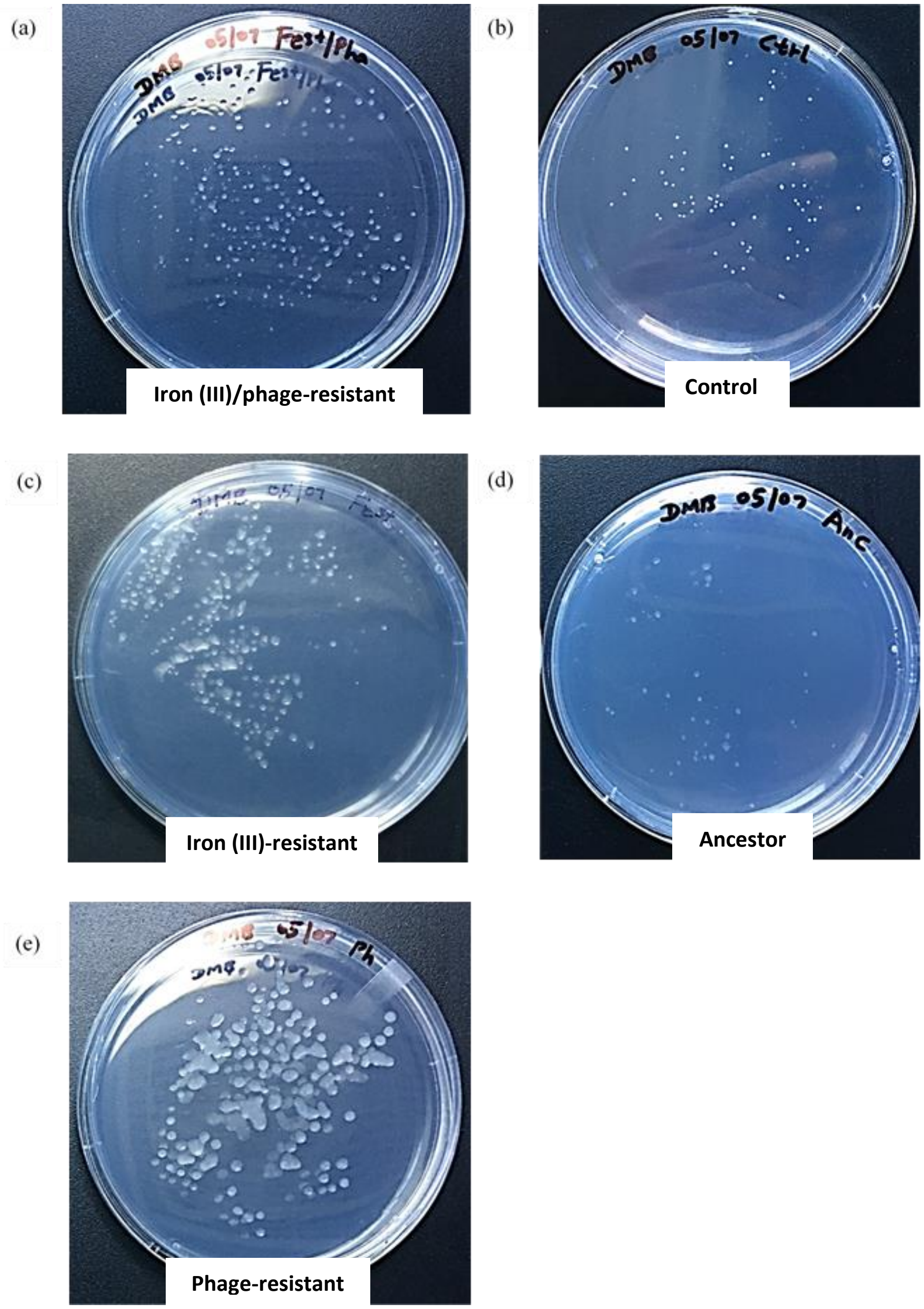

Table S1. Multiple comparisons of resistance of populations to lytic phage in a plaque assay. *The mean difference is significant at the 0.05 level

(I) Populations	(J) Populations	Mean difference (I-J)	Significance
Control	Iron (III)/phage	147.17*	<0.001
	Iron (III)	64.33*	<0.001
	Phage	147.17*	<0.001
	Ancestor	-50.17*	<0.001
Iron (III)/phage	Control	-147.17*	<0.001
	Iron (III)	-82.83*	<0.001
	Phage	0.00	1.000
	Ancestor	-197.33*	<0.001
Iron (III)	Control	-64.33*	<0.001
	Iron (III)/phage	82.83*	<0.001
	Phage	82.83*	<0.001
	Ancestor	-114.50*	<0.001
Phage	Control	-147.17*	<0.001
	Iron (III)/phage	0.00	1.000
	Iron (III)	-82.83*	<0.001
	Ancestor	-197.33*	<0.001
Ancestor	Control	50.17*	<0.001
	Iron (III)/phage	197.33*	<0.001
	Iron (III)	114.50*	<0.001
	Phage	197.33*	<0.001

Table S2. Multiple comparisons of dual resistant populations to lytic phage and iron (III) populations.
*The mean difference is significant at the 0.05 level

(I) Populations	(J) Populations	Mean difference (I-J)	Significance
Ancestor	Control	-13.20	0.870
	Iron (III)	-60.00*	0.001
	Iron (III)/Phage	-96.70*	0.000
	Phage	-75.30*	0.000
Control	Ancestor	13.20	0.870
	Iron (III)	-46.80*	0.001
	Iron (III)/Phage	-83.50*	0.000
	Phage	-62.10*	0.000
Iron (III)	Ancestor	60.00*	0.001
	Control	46.80*	0.011
	Iron (III)/Phage	-36.70	0.074
	Phage	-15.30	0.797
Iron (III)/phage	Ancestor	96.70*	0.000
	Control	83.50*	0.000
	Iron (III)	36.70	0.074
	Phage	21.40	0.530
Phage	Ancestor	75.30*	0.000
	Control	62.10*	0.000
	Iron (III)	15.30	0.797
	Iron (III)/Phage	-21.40	0.530

Table S3. (a) Position of minor polymorphisms (blue) in iron (III)/phage-resistant populations at day 35. (b) Annotation of genes mutated (blue- missense mutation; red- nucleotides changed) in iron (III)/phage-resistant populations at day 35.

(a)

Gene	Position	Mutation	FPh1	FPh4	FPh5	FPh6
dapB → / → *carA*	29,330	C→T	0.000	0.101	0.000	0.000
clpX → / → *lon*	458,790	IS186 (+) +6 bp :: Δ1 bp	0.000	0.283	0.000	0.252
ybfL →	737,627	C→A	0.000	0.079	0.100	0.000
tolA →	777,124	T→A	0.000	0.211	0.000	0.149
ycdU → / ← *serX*	1,097,158	C→T	0.000	0.000	0.000	0.115
racC ←	1,417,590	G→T	0.162	0.000	0.000	0.000
fliR → / → *rcsA*	2,023,823	C→T	0.000	0.321	0.000	0.268
rseX → / → *yedS*	2,033,742	G→T	0.000	0.000	0.135	0.000
wcaC ←	2,131,514	T→C	0.000	0.000	0.000	0.112
asmA ←	2,140,214	G→A	0.000	0.000	0.115	0.000
asmA ←	2,140,883	G→A	0.000	0.214	0.188	0.245
yejM →	2,286,084	T→G	0.122	0.000	0.000	0.000
rcsC ←	2,317,381	A→C	0.111	0.000	0.000	0.000
ygcW ←	2,900,146	G→T	0.000	0.000	0.133	0.000
gcvT ← / ← *ubiI*	3,050,867	G→A	0.000	0.000	0.140	0.000
gcvT ← / ← *ubiI*	3,050,938	C→A	0.000	0.100	0.000	0.000
gcvT ← / ← *ubiI*	3,050,951	T→A	0.000	0.000	0.133	0.000
rrsD ← / → *yrdA*	3,429,162	G→T	0.000	0.000	0.108	0.079
waaC →	3,796,802	IS3 (–) +4 bp :: +TC	0.000	0.187	0.000	0.180
waaS ←	3,805,057	C→T	0.000	0.101	0.013	0.000
waaP ←	3,805,170	G→T	0.000	0.093	0.119	0.000
sbp → / → *cdh*	4,109,912	A→G	0.000	0.000	0.105	0.000
rpoB →	4,182,820	C→T	0.000	0.268	0.000	0.199
rpoC →	4,187,356	+TCT	0.000	0.165	0.000	0.220
malE ← / → *malK*	4,246,678	A→C	0.000	0.000	0.102	0.000
adiY ← / ← *adiA*	4,338,025	C→A	0.000	0.000	0.000	0.100
adiY ← / ← *adiA*	4,338,081	C→A	0.000	0.000	0.110	0.000
adiY ← / ← *adiA*	4,338,093	C→A	0.000	0.000	0.000	0.113
adiY ← / ← *adiA*	4,338,109	C→A	0.000	0.188	0.201	0.201
rnr →	4,408,897	T→A	0.000	0.113	0.000	0.000
lptF →	4,486,776	Δ3 bp	0.000	0.000	0.113	0.000

(b)

Gene	Annotation
clpX → / → *lon*	intergenic (+90/-93)
clpX → / → lon	intergenic (+90/-93)
uxaB ← / ← *yneF*	intergenic (-127/+100)
yeaG →	E414K (GAA→AAA)
proQ ←	coding (57/699 nt)
fliR → / → *rcsA*	intergenic (+146/-144)
[gatR]–[fbaB]	IS3-mediated
yejM →	Q356* (CAG→TAG)
yejM →	S363* (TCG→TAG)
yejM →	coding (1439-1448/1761 nt)
rcsC ←	coding (81-85/2850 nt)
ypjF → / ← *ypjA*	intergenic (+200/+164)
rpoS ←	G126V (GGG→GTG)
rpoS ←	L125Q (CTG→CAG)
rpoS ←	E42* (GAA→TAA)
rpoS ←	coding (230/993 nt)
waaC →	coding (41/960 nt)
waaC →	coding (189-191/960 nt)
rpoB →	G1189V (GGT→GTT)
rpoC →	K395E (AAA→GAA)
rpoC →	M725L (ATG→CTG)
pgi → / → *yjbE*	intergenic (+275/-224)
yjbH →	Y102S (TAT→TCT)
yjbH →	W147* (TGG→TAG)
yjbH →	coding (1810-1814/2097 nt)
adiY ← / ← *adiA*	intergenic (-180/+145)
hfq →	P64L (CCG→CTG)

Stop codons are symbolized as *

Table S4. (a) Position of minor polymorphisms (blue) in iron (III)-resistant populations at day 35. (b) Annotation of genes mutated (blue- missense mutation; red- nucleotides changed) in iron (III)-resistant populations at day 35.

(a)

Gene	Position	Mutation	Fe1	Fe2	Fe5	Fe7
rpoB →	4,183,154	G→C	0.000	0.000	0.283	0.000
dctA ← / ← *yhjK*	3,683,472	G→A	0.210	0.000	0.000	0.000
rpoB →	4,183,379	C→T	0.000	0.000	0.000	0.112
rpoB →	4,182,809	C→A	0.165	0.000	0.000	0.000
yeaH →	1,869,484	G→C	0.000	0.183	0.000	0.000
yeaH →	1,869,699	G→T	0.000	0.000	0.179	0.000
cspC ←	1,907,307	IS2 (–) +5 bp	0.117	0.000	0.000	0.000
rseX → / → *yedS*	2,033,756	G→T	0.174	0.000	0.000	0.160

(b)

Gene	Annotation
rpoB →	R637P (CGT→CCT)
dctA ← / ← *yhjK*	intergenic (-25/+158)
rpoB →	S712F (TCC→TTC)
rpoB →	S522Y (TCT→TAT)
yeaH →	R177P (CGC→CCC)
yeaH →	A249S (GCA→TCA)
cspC ←	coding (125-129/210 nt)
rseX → / → *yedS*	intergenic (+17/-295)

Table S5. (a) Position of minor polymorphisms (blue) in phage-resistant populations at day 35. (b) Annotation of genes mutated (blue- missense mutation; red- nucleotides changed) in phage-resistant populations at day 35.

(a)

Gene	Position	Mutation	Ph2	Ph3	Ph4
yejM →	2,285,463	C→A	0.000	0.000	0.498
yejM →	2,285,814	Δ10 bp	0.000	0.000	0.401
rcsC ←	2,319,792	IS*2* (–) +5 bp	0.000	0.479	0.000
rpoS ←	2,867,428	C→A	0.183	0.000	0.000
rpoB →	4,184,810	G→T	0.258	0.000	0.000
adiY ← / ← *adiA*	4,338,109	C→A	0.210	0.000	0.000
hfq →	4,400,478	C→T	0.000	0.000	0.401

(b)

Gene	Annotation
yejM →	S363* (TCG→TAG)
yejM →	coding (1439-1448/1761 nt)
rcsC ←	coding (81-85/2850 nt)
rpoS ←	E42* (GAA→TAA)
rpoB →	G1189V (GGT→GTT)
adiY ← / ← *adiA*	intergenic (-180/+145)
hfq →	P64L (CCG→CTG)

Stop codons are symbolized as *
